# Parametric Optimization of VLM Panel Discretization Using Bio-Inspired Crayfish and Aquila Algorithms Coupled with Hybrid RSM-Based Ensemble Machine Learning Surrogate Models: A Case Study

**DOI:** 10.3390/biomimetics11030204

**Published:** 2026-03-11

**Authors:** Yüksel Eraslan, Esmanur Şengün

**Affiliations:** 1 Aerospace Engineering Department, Tarsus University, Mersin 33400, Türkiye; 2 School of Graduate Studies, Tarsus University, Mersin 33400, Türkiye; esmasngn05@gmail.com

**Keywords:** vortex lattice method, panel discretization, hybrid response surface method, ensemble machine learning, aquila optimization, crayfish optimization

## Abstract

Fast and reliable aerodynamic predictions are crucial in the early phases of aircraft design, where a quick assessment of various configurations is required. In this context, the Vortex Lattice Method (VLM) is widely adopted due to its computational efficiency; however, its predictive accuracy is highly sensitive to panel discretization strategies, which are often determined heuristically. This study proposes a bio-inspired optimization framework for VLM panel discretization and evaluates it through a systematic case study on a representative wing geometry. A grid-convergence analysis was initially carried out to ensure solution independence across various spanwise-to-chordwise panel ratios. Subsequently, a novel Hybrid Response Surface Methodology (HRSM), integrating Box–Behnken and Central Composite experimental designs, was employed to enable a more comprehensive exploration of the factor space while quantifying the effects of clustering parameters at the leading-edge, trailing-edge, root, and tip regions of the wing. The HRSM dataset was further utilized to train Ensemble Machine-Learning surrogate models, which were coupled with bio-inspired Crayfish and Aquila optimization algorithms, alongside a classical Genetic Algorithm (GA) as a performance benchmark, to identify the optimal discretization strategy and to enable a comparative assessment of their convergence behavior and robustness against the numerical noise of the ensemble-based landscape. Compared to base (i.e., uniform) panel distribution, the optimally clustered discretization enhanced overall aerodynamic prediction accuracy by approximately 33%, particularly at low angles of attack, while maintaining robust performance at higher angles. Both algorithms converged to similar minima; however, the Aquila algorithm achieved higher solution consistency, whereas the Crayfish algorithm exhibited greater dispersion despite faster convergence, revealing a multimodal optimization landscape. The variance decomposition revealed that trailing-edge clustering dominated aerodynamic accuracy at low angles of attack, contributing up to 90% of the total variance, whereas tip clustering became increasingly influential at higher angles, exceeding 30%, highlighting the need for adaptive discretization strategies to ensure reliable VLM-based aerodynamic analyses.

## 1. Introduction

The modern aircraft design process typically follows a structured, multi-phase approach that includes conceptual, preliminary, and detailed design stages. During the conceptual design, multidisciplinary trade-off analyses are conducted to establish the baseline aircraft configuration, including aerodynamics, structural characteristics, propulsion systems, and weight estimation [1]. Within this multidisciplinary framework, aerodynamics plays a critical role, and the accurate prediction of lift and drag characteristics is essential, as these characteristics directly affect stability, controllability, and overall flight performance. The Vortex Lattice Method (VLM) is commonly used for fast, cost-effective aerodynamic predictions by discretizing lifting surfaces into a finite number of vortex panels. However, the method’s predictive accuracy is highly sensitive to the distribution and clustering of panels across the geometry. Optimally adjusted panel discretization can improve the accuracy of aerodynamic predictions and enhance the robustness of VLM, supporting more reliable trade-off analyses while preserving the computational efficiency required for early-stage conceptual design, where 70–80% of an aerial vehicle’s total life-cycle cost is determined.

The number and arrangement of VLM panels are typically determined using heuristic or experience-driven methods, such as uniform distributions for preliminary assessments, or analytical spacing functions, including linear, cosine, semi-cosine, and full-sine distributions, to resolve spanwise and chordwise variations in circulation distributions [2,3,4]. Besides these basic techniques, structured algebraic spacing strategies, such as geometric progression and hyperbolic tangent stretching functions, are frequently employed to cluster panels near the leading edges and wing tips. In practice, geometric quality considerations (e.g., panel aspect ratio and chordwise–spanwise resolution balance) are also manually tuned to improve numerical stability and convergence behavior [5]. However, these traditional methodologies exhibit significant limitations. Deterministic spacing functions are inherently static; they require manual parameter tuning and fail to account for dynamic variations in circulation behavior across different angles of attack or operating conditions [2]. Furthermore, traditional discretization methods treat chordwise and spanwise spacing as uncoupled variables. This oversight often yields high-aspect-ratio panels that introduce numerical noise and lead to poorly conditioned aerodynamic influence matrices. Although localized grid-sensitivity adjustments may force solution consistency in specific regions, these rigid grids ultimately fail to adapt to steep circulation gradients at the wing tips under varying flight conditions. Consequently, the literature still lacks a comprehensive investigation and structured optimization framework that explicitly treats discretization parameters as coupled, interacting variables. Recent efforts to automate VLM panel discretization have focused on adaptive discretization techniques and gradient-based optimization algorithms [6,7]. While adaptive discretization refines grids dynamically based on local flow gradients, it greatly increases computational effort, thereby diminishing the main benefit of VLM in quick conceptual design. On the other hand, traditional gradient-based optimization methods often struggle with nonlinear, multimodal, and sometimes discontinuous aerodynamic influence matrices, often converging to local optima rather than the global optimum. To overcome the high computational demands of adaptive panel discretization and the challenge of local minima in gradient-based methods, surrogate-based optimization approaches have become more popular.

Response Surface Methodology (RSM) is a robust statistical and mathematical technique used to develop, improve, and optimize processes in which a response of interest is influenced by several variables, like the discretization parameters. The method has been widely adopted in aerodynamic studies to construct efficient surrogate models for complex optimization problems, such as investigating non-linear fan blade geometries [8], enhancing the lift-to-drag ratio of UAV morphing flaps [9], and improving the aerodynamic efficiency of wing configurations [10]. To maximize the predictive accuracy of these models, various experimental designs and sampling architectures have been explored. For instance, Latin Hypercube Sampling (LHS) has been used to develop airfoil and engine nacelle models [11,12], while comparisons have shown that RSM is particularly effective for optimization problems with lower data complexity than Artificial Neural Networks [13]. In general, the Box–Behnken Design (BBD) or the Central Composite Design (CCD) is commonly preferred for modeling second-order response surfaces [14,15]. In this framework, Analysis of Variance (ANOVA) also serves as a critical diagnostic tool, validating the statistical significance of the RSM models and confirming that the observed variations in aerodynamic responses are reliably driven by the chosen discretization parameters. Although ANOVA provides valuable insights into parameter sensitivity, finding the optimal panel configuration requires exploring a complex, nonlinear, and multimodal design space. This involves handling interdependent spanwise and chordwise clustering variables, which can be difficult for standard optimization methods.

Traditional gradient-based or deterministic optimization techniques often struggle with complex, multimodal, and black-box objective functions, especially when they involve mixed continuous and discrete design variables [16,17]. In contrast, nature-inspired metaheuristic algorithms offer derivative-free global search capabilities and demonstrate high robustness in complex engineering design spaces. Their population-based approach facilitates a good balance between exploration and exploitation, making them especially suitable for surrogate-assisted optimization frameworks [18]. Therefore, bio-inspired algorithms have become increasingly preferred in up-to-date studies on aerodynamic and multidisciplinary design optimization problems. For instance, well-established metaheuristics such as Genetic Algorithms (GA) and Particle Swarm Optimization (PSO) have been extensively and successfully applied to address highly nonlinear aerodynamic challenges, including airfoil shape optimization, wing planform design, and drag reduction [19,20,21]. Building on the proven success of these classical nature-inspired methods, researchers are continuously exploring next-generation algorithms that offer superior convergence rates and better avoidance of local optima in complex surrogate landscapes. In the recent literature, the recently introduced bio-inspired Aquila Optimizer (AO) and Crayfish Optimization Algorithm (COA) have emerged as powerful tools in this context, with various hybrid strategies developed to balance exploration and exploitation. The AO simulates the eagle’s social hunting stages, transitioning from high-altitude exploration (soaring and contour flight) to precise low-altitude maneuvers for exploitation. Similarly, the COA mimics the environmental adaptations of crayfish, such as heat-avoidance and food-competition behaviors, to balance randomized exploration with localized search. These algorithms have been introduced very recently to the literature; however, the limited studies show that while simplified or enhanced versions of AO have been developed to improve convergence speed and robustness in engineering problems [22,23,24], Crayfish-based hybrid models, often supported by differential evolution, specular reflection methods, or vertical crossover operators, demonstrate superior stability and accuracy in complex design spaces [25,26,27,28]. Furthermore, integrating these algorithms with surrogate models has proven successful across diverse applications, ranging from turbine blade disc optimization [27] to predicting material wear [26]. However, the relative performance of such metaheuristic solvers depends heavily on the nature of the objective function and the problem’s specific constraints.

In this study, a systematic optimization framework is proposed to enhance the aerodynamic predictive accuracy of VLM by adjusting panel discretization, demonstrated through a case study on a representative wing geometry. The methodology incorporates a Hybrid Response Surface Methodology (HRSM) to enable a broader exploration of the discretization factor space and to support second-order response modeling. The hybrid dataset is also utilized to train Ensemble Machine Learning (EML) surrogate models, establishing a high-fidelity predictive bridge. This integrated structure is then coupled with two state-of-the-art metaheuristics, AO and COA, alongside a traditional GA as a baseline benchmark, to identify the most effective panel discretization within a comparative perspective. A comprehensive ANOVA is conducted to examine how clustering parameters in the leading-edge, trailing-edge, root, and tip regions influence the lift and drag coefficients predicted by VLM.

This research pioneers a novel approach for multidisciplinary design optimization in aerodynamics by combining advanced statistical, learning, and biomimetic techniques within a unified architecture. While VLM panel discretization is inherently an aerodynamic problem, the multimodal and noise-sensitive nature of the surrogate-based optimization landscape demands advanced search strategies. In this regard, this study bridges aerodynamics and biomimetics by investigating how distinct biological behaviors navigate this complex design space when modeled mathematically. Rather than using bio-inspired algorithms just as ‘black-box’ tools, this research examines how the deterministic, targeted hunting strategies of the Aquila and the temperature-driven, dispersed foraging behaviors of the Crayfish influence their convergence and robustness compared to traditional evolutionary mechanics. This biological inspiration is crucial to the study’s scientific insight, offering the analytical perspective needed to understand why certain search behaviors succeed or fail in a complex aerodynamic environment. For example, modeling Aquila’s precise hunting techniques explains its capacity to optimize highly sensitive parameters, while the Crayfish’s widespread foraging demonstrates the multimodal characteristics of the design space. By explicitly linking algorithmic performance to their biological inspirations, the study offers important insights into how biomimetic mechanisms address the exploration-exploitation trade-off in complex, multidisciplinary engineering problems.

The main original contributions of this study can be summarized as follows:

(1) The effects of panel discretization and clustering on VLM aerodynamic predictions are systematically examined via RSM and ANOVA to quantify factor significance and interaction effects, and methodological insights are presented that may serve as procedural references for future panel discretization and optimization studies on similar wing topologies.
(2) The Box–Behnken and Central Composite experimental designs are combined into a single dataset to facilitate Hybrid RSM analysis and ensemble machine-learning surrogate modeling of aerodynamic responses. This novel integration approach enables broader exploration of the factor space and demonstrates the effectiveness of the combined DOE–ML approach.
(3) The bio-inspired AO and COA are compared in their effectiveness for VLM panel clustering optimization within a specific aerodynamic design scenario for the first time, offering insights into their robustness, convergence behavior, and solution variability. Moreover, the study clearly links their unique biological inspirations (the deterministic, targeted hunting of the Aquila and the dispersed, temperature-dependent foraging of the Crayfish) to their real algorithmic effectiveness in exploring a multimodal, noise-sensitive aerodynamic environment. By incorporating a traditional GA as a performance baseline, the study provides a thorough assessment of these modern metaheuristics in terms of robustness, convergence patterns, and resistance to numerical noise in ensemble-based surrogate landscapes.
(4) The findings indicate that for the investigated case study, the optimal panel discretization varies with the angle of attack, and lift and induced drag favor different clustering configurations, highlighting trade-offs and supporting adaptive, balanced discretization strategies for consistent aerodynamic performance.
(5) The potential of systematic panel discretization optimization is revealed to achieve significant improvements in VLM aerodynamic prediction accuracy, which is quantitatively validated with experimental data.

## 2. Materials and Methods

**Figure 1:**
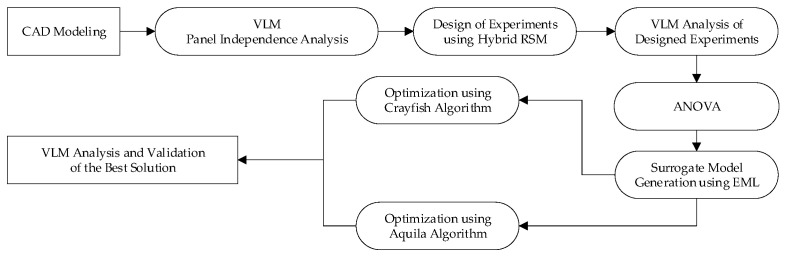
Block diagram of the optimization process.

The initial step of the process in this study relies on the design of experiments, generating various combinations of design parameters to investigate. The combinations were then analyzed using the VLM in VSPAERO, and an ANOVA was conducted to assess the effects of discretization parameters on the resulting lift and drag coefficients. The collected datasets were subsequently used to develop surrogate models within an EML framework, combined with the bio-inspired Crayfish and Aquila optimization algorithms, to determine the optimal number of panels and the clustering options to improve accuracy relative to experimental data. The workflow block diagram is shown in Figure 1. This section first introduces the wing geometry under investigation, followed by a detailed description and validation of the numerical, statistical, and optimization methodologies employed.

### 2.1. Vortex Lattice Method

OpenVSP (Open Vehicle Sketch Pad) is a parametric design tool developed by the National Aeronautics and Space Administration (NASA), primarily used for geometric modeling of aerospace vehicles [29]. The software also includes an integrated aerodynamic solver, VSPAERO, capable of providing rapid and reliable potential-flow predictions, particularly for subsonic flight conditions, and of performing the VLM or the Panel Method (PM) [30]. In this study, the VSPAERO (version 3.42.3) module was employed for VLM analyses, facilitating the determination of the linear aerodynamic characteristics of the wing geometry as described in the following section.

VLM is a computational method for solving steady, incompressible, and inviscid (potential) flow equations and, correspondingly, for predicting the lift distribution, induced drag, and pitching moments of finite wings, especially at low Reynolds numbers and moderate angles of attack. The approach discretizes the lifting surfaces into a grid of horseshoe- or ring-shaped vortices to determine the unknown circulation strengths. In VSPAERO, horseshoe vortices are employed to solve the linear system derived from the No-Penetration Condition, which states that the velocity component normal to the lifting surface must be zero at a specific control point *i* on each panel [31]. The initial equation is given as

(1) $$\sum_{j}^{N}a_{ij}\Gamma_{j}=V_{\infty }\cos(a_{i})$$

where $a_{ij}$ are the aerodynamic influence coefficients, Γ*_j_* is the vortex circulation strength, *V_∞_* is the freestream velocity, α*_i_* is the local angle of attack, and *i* and *j* are the index numbers referring to control points and vortices, respectively. Once the circulation strengths have been calculated, the lift contribution, Δ*L_j_*, of each vortex segment is computed via the Kutta–Joukowski Theorem,

(2) $$\Delta L_{j}=\rho V_{\infty }\Gamma_{j}\Delta s_{j}$$

where *L* is the lift force, *ρ* is the density, *s_j_* is the panel width in the spanwise direction, and *N* is the total number of vortex elements. The total lift, *L*, is the summation of the panel lift contributions. Subsequently, the lift coefficient, *C_L_*, is calculated using the following definition,

(3) $$C_{L}=\frac{\sum_{j=1}^{N}\Delta L_{j}}{\frac{1}{2}\rho V_{\infty }^{2}S}$$

where *S* is the wing area, and *V_∞_* is the freestream velocity. The total drag is calculated by summing the induced drag coefficient, *C_Di_*, and the parasite drag coefficient, *C_D_*_0_, as given in Equation (4).

(4) $$C_{D}=C_{D_{0}}+C_{D_{i}}$$

The induced drag coefficient is determined from the obtained lift coefficient, typically using the standard formulation derived from lifting-line theory,

(5) $$C_{D_{i}}=\frac{C_{L}^{2}}{\pi Ae}$$

where *A* is the wing aspect ratio, and *e* is the Oswald efficiency factor of the wing. The parasite drag coefficient is often estimated empirically, accounting for form drag and skin-friction drag. One common approach is the Torenbeek statistical method [32], which is crucial for converting the potential flow solution into a realistic aerodynamic performance prediction that correlates the parasite drag coefficient with component wetted areas, component-specific form factors, and surface roughness factors. At low speeds, the flow is treated as incompressible, and the effects of fluid compressibility (e.g., wave drag) are neglected; parasite drag is dominated by skin friction and form drag and can therefore be assumed to be independent of angle of attack. VSPAERO solves the inviscid potential flow equations, incorporating viscous effects indirectly by using two-dimensional viscous polar data for airfoils as input to three-dimensional analyses, thereby accounting for real-fluid effects.

#### 2.1.1. Wing Geometry and Discretization Parameters

**Figure 2:**
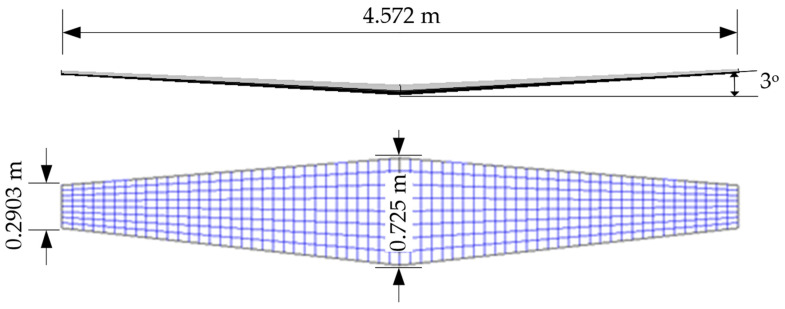
Dimensional properties, front view (**upper**), and top view (**lower**) of the wing geometry discretized into panels.

The wing geometry, featuring a NACA 64-210 airfoil section and a 2-degree washout, was modeled in OpenVSP (version 3.42.3) with no quarter-chord sweep, and the dimensions are shown in Figure 2, as specified in the National Advisory Committee for Aeronautics technical note [33].

VSPAERO offers user-defined discretization options, in which the parameters Num_U and Num_W denote the number of nodes in the chordwise (longitudinal) and spanwise (lateral) directions, respectively. If these parameters are equal, the span-wise to chord-wise panel ratio (SCPR) yields 2, corresponding to the grid distribution shown in Figure 2. The additional discretization options affecting the accuracy and convergence of the solution are the leading-edge clustering (LEC), trailing-edge clustering (TEC), root cluster (RT), and tip cluster (TC) parameters, which enable adjustment of panel density within the desired wing section. Consequently, the discretization parameters are set as Num_U, Num_W, LEC, TEC, RT, and TC to examine their effects on reducing numerical errors and improving the accuracy of aerodynamic estimates.

#### 2.1.2. Panel Independence Analysis

In order to ensure that the number of panels does not affect aerodynamic results, a panel-independence analysis is conducted before investigating clustering options. The SCPR, as given in Equation (6), was also considered with the number of panels.

(6) $$\mathrm{SCPR}=\frac{2(\mathrm{Num}\_U-1)}{\left(\mathrm{Num}\_W-1\right)}$$

**Figure 3:**
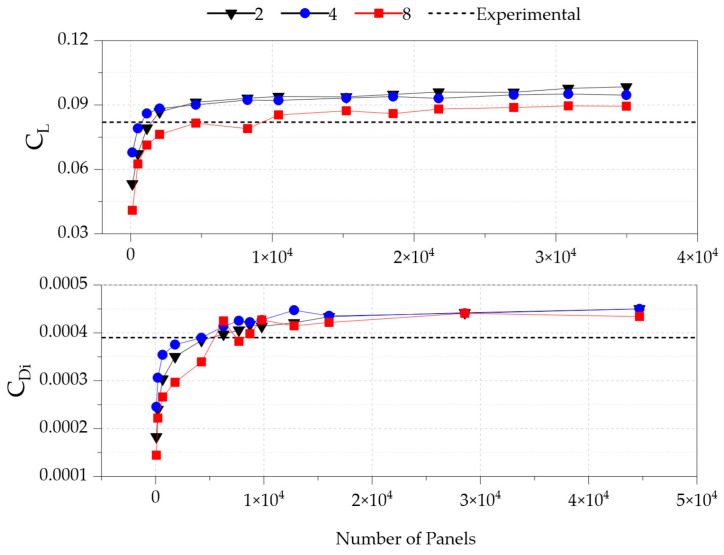
Panel independence results for lift and induced drag coefficients at a 0-degree angle of attack for SCPR of 2, 4, and 8.

The number of panels was increased in three groups, yielding SCPR values of 2, 4, and 8, with an appropriate number of spanwise and chordwise nodes. The analyses were conducted in VSPAERO at a 0° angle of attack and an airspeed of 0.17 Mach, corresponding to a Reynolds number of 4.4 × 10^6^. Since the constant parasite-drag component is estimated using the aforementioned statistical method, which is independent of the number of panels, the results are presented only for the lift and induced-drag coefficients in Figure 3, compared with the experimental data reported in [33].

**Figure 4:**
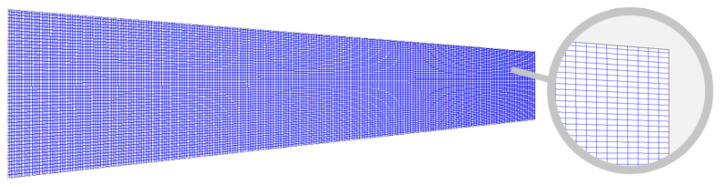
Top view of the half-wing geometry with 21,736 panels without clustering.

The figure clearly shows that around 20,000 panels are adequate for all groups to achieve consistent results regardless of the total number of panels. However, discretization using an SCPR of 8 produced the most stable results and aligned most closely with the experimental data, in good agreement with the previous study by Sheridan et al. [5]. The differences compared to wind-tunnel data were 7.46% for the lift coefficient and 6.30% for the induced drag coefficient, achieved using proper discretization with 210 spanwise nodes and 105 chordwise nodes, which resulted in 418 spanwise panels and 52 chordwise panels, as shown in Figure 4.

### 2.2. Hybrid Response Surface Methodology

RSM is a Design of Experiments (DOE) technique for modeling and analyzing engineering problems in which a response is affected by multiple input variables, with the primary objective of optimization [34]. Recent comparative studies in engineering optimization demonstrate that RSM remains a superior approach for reducing computational cost in high-fidelity simulations compared to one-factor-at-a-time (OFAT) methods [35]. The method uses quantitative data from appropriately designed experiments to determine and define the complex relationship between process parameters and the desired output. The most popular methods include Box–Behnken Design (BBD) and Central Composite Design (CCD), both of which are suitable for developing second-order models. CCD is preferred for its robust exploration of the experimental space’s boundaries via axial points and is appropriate when the optimum may lie at the edge or when high prediction accuracy is required. However, BBD offers a more cost-effective solution with fewer runs and is especially useful when corner-point experiments are more difficult.

In the OpenVSP framework, the four clustering parameters (LEC, TEC, RC, and TC) dictate the spatial discretization of the VLM grid along the chordwise and spanwise directions. Physically, these parameters control panel density in critical regions characterized by complex flow phenomena. LE and TE clustering are crucial for accurately capturing the sharp pressure gradients at the leading edge’s suction peak and the wake behavior driven by the Kutta condition at the trailing edge. Likewise, Root and Tip clustering effectively model three-dimensional flow phenomena, especially wingtip vortex formation, which influences the induced drag coefficient.

The design-parameter constraints for all variables are set to 0.1–1.5 for BBD and 0.575–1.525 for CCD (with an axial distance of α = 2). The upper limit of 1.5 provides the optimization algorithm with the flexibility to explore coarsened distributions. However, establishing the lower bounds required careful attention, given the theoretical nature of DOE. Theoretically, BBD evaluates points only at the center and the midpoints of the edges of the process space, and contains all design points within the specified bounds, avoiding extreme cubic vertices or extrapolated points. Thus, the lower bound selection of 0.1 captures extremely high-gradient aerodynamic clustering without the risk of generating non-physical zero or negative values. Conversely, CCD is fundamentally structured to maintain rotatability and capture quadratic curvature by generating axial (star) points that extrapolate beyond the defined factorial block by a distance of α = 2. Predicting this theoretical outward expansion, the parameter limits for CCD were deliberately reverse-engineered and established within a range of 0.575–1.525. This proactive adjustment ensures that even the most extreme extrapolated axial points safely drop to around the 0.1 aerodynamic limit without crossing into the non-physical negative domain, thus maintaining the geometric integrity of the spatial grid and preventing mathematical singularities in the VLM solver.

Consequently, the BBD included three center points, while the CCD employed five center points to estimate experimental error and assess model curvature. The design matrices generated in Minitab (version 22.0) are shown in Table 1. The designed experiments are presented in Tables S1 and S2.

### 2.3. Surrogate Model Generation via Ensemble Machine Learning Approach

The most common surrogate models in the literature include the polynomial RSM, kriging, and machine learning (ML) techniques. ML methods, particularly ensemble techniques such as Random Forests and Gradient Boosting, achieve higher predictive accuracy by modeling complex, nonlinear, high-dimensional interactions while also reducing the overfitting risk common to single models. ML frameworks cover supervised, unsupervised, and reinforcement learning.

This study targets explicitly a supervised learning regression task to predict continuous values of lift and induced drag coefficients. The data points generated in Table 1 using RSM are intended to enable robust exploration of the multidimensional parameter space. The dataset comprises lift and induces drag coefficients analyzed at angles of attack of 0° and 5° for each design point. To enhance prediction accuracy for complex data patterns, an Ensemble Learning Approach was adopted. This powerful meta-algorithm combines the outputs of multiple base models, or weak learners, to produce more robust and accurate predictions. The Ensemble model for our case comprises the input feature vector *X*, defined by four geometric variables, and the output response vector *Y*, representing the lift and induced drag coefficients, as described in Equations (7) and (8).

(7) $$X=\left[TEC,\;LEC,\;RC\;,TC\right]$$

(8) $$Y={\left[C_{L},C_{D_{i}}\right]}^{T}$$

An ensemble predictor is obtained as in Equation (9), where *M* denotes the total number of base models, *α_m_* denotes the importance weight assigned to the *m*th base learner, and *f_m_* denotes the prediction function at stage *m*, with *m* ranging from 1 to *M*.

(9) $$F(X)=\sum_{m=1}^{M}\alpha_{m}f_{m}(X)$$

The approach mathematically addresses bagging methods such as Random Forests, which primarily reduce the overall variance of the predictor by averaging uncorrelated errors from individual models. In contrast, boosting methods such as LSBoost focus on decreasing bias by training new models to correct the systematic errors (residuals) of previous models. LSBoost employs functional gradient descent to minimize the Mean Squared Error (MSE) loss function *L*, as defined in Equation (10).

(10) $$L(F)=\sum_{i=1}^{N}\frac{1}{2}(y_{i}-F(X_{i}){)}^{2}$$

At each stage *m*, a new base learner, *f_m_*, is trained on the pseudo-residuals *r_im_*. For LSBoost, the pseudo-residual exactly matches the true residual of the previous ensemble approximation, as shown in Equation (11).

(11) $$r_{im}=y_{i}-F_{m-1}(x_{i})$$

The constructed framework offers notable benefits over conventional approaches by combining predictions from multiple weak learners to effectively manage the bias-variance trade-off. LSBoost iteratively minimizes the least-squares loss by fitting each new learner to the pseudo-residuals of the current ensemble, rather than using the Polynomial Response Surface Method (PRSM), which relies on fixed functional forms. This process reduces model bias and captures complex high-order interactions in the input space, ensuring accurate predictions.

The ensemble architecture employed regression trees as base learners, with carefully tuned hyperparameters for optimal performance: 500 learning cycles (trees) and a learning rate of 0.1. The minimum leaf size was set to 5, which limits the maximum depth of each regression tree to prevent overfitting to local noise. Surrogate splits were enabled to enhance split quality. Additionally, to thoroughly assess the reliability of the LSBoost algorithm and prevent biases from a single random data split, a 10-fold Cross-Validation (CV) process is incorporated into the modeling phase. This approach systematically divides the entire dataset, repeatedly applying a 90:10 training-to-testing split across ten different folds. The performance metrics obtained from this CV process, which confirm the models’ ability to generalize and their structural stability, are fully presented and analyzed in Section 3.

### 2.4. Optimization Algorithms

The panel independence analysis resulted in a panel discretization with an SCPR of 8, without clustering. However, applying suitable clustering options can enhance the accuracy of the analyses. To find the optimal clustering configuration and improve fidelity, this study employs advanced, nature-inspired algorithms based on Crayfish and Aquila integrated with a pretrained EML surrogate model. Additionally, a traditional GA is implemented as a baseline to thoroughly evaluate the performance of these modern metaheuristics, especially considering the numerical noise and step-wise discontinuities present in ensemble-based surrogate landscapes [36]. A continuous GA with a population size of 30 and up to 500 iterations was used to objectively assess the relative robustness, stability, and exploration efficiency of the proposed AO and COAs.

The optimization process is governed by a composite objective function, *f*(*X*), that minimizes the total deviation from performance targets across multiple operating conditions, as given in Equation (12). This function is defined as the sum of the normalized relative errors denoted by *e*. Specifically, *eC_Lα_* and *eE_α_* represent the relative deviations of the predicted values from the target values for the lift coefficient and the aerodynamic performance (*E*), which is defined as the lift-to-drag ratio (L/D), respectively, at the two angles of attack, α = 0° and α = 5°. The aerodynamic performance is evaluated using the lift-to-drag ratio (L/D). While the surrogate models directly predict *C_L_* and *E*, the induced drag coefficient is analytically derived from Equation (4) as a secondary parameter using the estimated parasite drag coefficient.

(12) $$\min f\left(X\right)=\left(eC_{L,\;0^{{}^{\circ}}}+eE_{L,\;0^{{}^{\circ}}}\right)+\left(eC_{L,\;5^{{}^{\circ}}}+eE_{L,\;5^{{}^{\circ}}}\right)$$

The optimization task is conducted subject to the inequality constraints given in Equation (13), which define the box constraints of the feasible design space for the decision variables, ensuring that each geometric parameter remains within the physical bounds expressed.

(13) $$g\left(X\right):0.1\leq x_{i}\leq 1.9$$

#### 2.4.1. Crayfish Algorithm

A crayfish (crawfish, crawdad, or mudbug) is a freshwater crustacean that looks like a miniature lobster. There are more than 600 species found across all continents except Antarctica and Africa. The crayfish responds strongly to environmental changes, especially temperature in nature. In hot summer conditions, they often seek shelter in caves or shaded areas to conserve energy and survive; when temperatures are optimal, they increase their foraging activity. If several crayfish find a dependable food source, they might compete or feed collaboratively, balancing the need to explore new territories by exploiting familiar resources.

The COA is a nature-inspired, population-based metaheuristic that mimics crayfish foraging and survival behaviors, proposed by Jia et al. [37]. COA is characterized by multi-stage transition logic that mimics crayfish’s adaptation to environmental variables, whereas other traditional algorithms rely on a single movement strategy. The algorithm is particularly effective at switching between exploration and exploitation in response to environmental factors, including heat and food competition.

In our case, the optimal set of clustering options is determined from the design vector given in Equation (7). The initial population is defined as given in Equation (14), which represents a stochastically generated initial VLM paneling configuration. Instead of allowing each algorithm to generate its own random starting population, a single “local initialization” was pre-initialized and applied to ensure a fair and direct comparison between the two optimization algorithms [38]. To improve search efficiency, an initial population of *N* individuals is generated using a narrow Gaussian distribution centered on a reference clustering configuration specified in Equation (15), where σ denotes the standard deviation and is set to 0.01 to ensure the search starts from a known baseline while preserving sufficient initial diversity.

(14) $$X_{start}=\left[TEC_{init},\;LEC_{init},\;RC_{init},\;TC_{init}\right]$$

(15) $$X_{i}=X_{start}+rand(N,\dim)\cdot \sigma$$

The algorithm uses a stochastic temperature parameter, *T*, to determine whether to pursue global exploration for new clustering configurations or local refinement of the best-known configuration. If this value exceeds 30, indicating summer behavior, the algorithm triggers reproductive behaviors, and maps to exploration and movement toward the global best to identify the most fertile search areas. If the value is below 30, the foraging phase predominates, and the crayfish focuses on finding food in its immediate vicinity, which the algorithm translates into refining the specific VLM panel clustering options. In the summer phase, a time-dependent step factor *p* is defined in Equation (16) and used in Equation (17), where *t* denotes the number of iterations, *T_max_* denotes the maximum number of iterations, rand denotes uniformly distributed pseudorandom numbers between 0 and 1, *X_new_* is the candidate, and *X_best_* is the global best solution.

(16) $$p=2\cdot rand\cdot (\frac{t}{T_{\max}})$$

(17) $$X_{new}=X_{i}+p+(X_{best}-X_{i})$$

In the foraging phase, the algorithm performs a localized search to fine-tune the clustering ratios using Equation (18).

(18) $$X_{new}=X_{i}-(X_{best}-X_{i})\cdot rand$$

#### 2.4.2. Aquila Algorithm

Aquila is a well-known bird of prey recognized for its agility and diverse hunting techniques in various environments and serves as the genus name for several eagle species. Inspired by nature, the AO Algorithm is a high-performance, gradient-free metaheuristic originally proposed by Abualigah et al. [39] that mimics four distinct hunting behaviors of the Golden Eagle (*Aquila chrysaetos*) to tackle complex optimization problems. These behaviors include high-altitude soaring with a vertical stoop for global exploration, contour flight with short glide attacks to pinpoint targets, low-level descents to concentrate on the prey’s immediate vicinity, and a final walking-and-grabbing move for accurate capture. By converting these natural actions into stochastic operators, AO effectively balances exploration of the search space with exploitation of promising regions and employs four distinct search operators to maintain a robust balance between global exploration and local exploitation.

In this study, the AO is employed to optimize the design vector specified in Equation (7), starting from the stochastically generated clustering configuration based on COA specified in Equation (14). The search process proceeds through two primary stages: exploration and exploitation, controlled by the current iteration *t* and a random threshold *r*. During the exploration stage (*t* ≤ 2/3*T_max_*), if *r ≤* 0.5, the algorithm performs Expanded Exploration by soaring high to identify the prey area as given in Equation (19),

(19) $$X_{1}(t+1)=X_{best}(t)\times (1-\frac{t}{T_{\max}})+(X_{mean}(t)-X_{best}(t)\times rand)$$

where *X_mean_* is the average position of the current population. When *r ≥* 0.5, the algorithm switches to Narrow Exploration, utilizing contour flight and Lévy flight (*L*) to simulate a stochastic search around a selected target, as given in Equation (20),

(20) $$X_{2}(t+1)=X_{best}(t)\times L+X_{rand}(t)+(rand-0.5)\times (UB-LB)$$

where *UB* and *LB* refer to upper and lower bounds of the variables [40]. In case of *r <* 0.5, Expanded Exploitation is executed via a low-flight descent to prepare for the attack as given in Equation (21),

(21) $$X_{3}(t+1)=(X_{best}(t)-X_{mean}(t))\times \alpha -rand+((UB-LB)\times rand+LB)\times \delta$$

where the exploitation parameters *α* and *δ* are fixed at 0.1. For the final Narrow Exploitation for *r ≥* 0.5, the eagle performs a precise swoop and grab as given in Equation (22),

(22) $$X_{4}(t+1)=QF\cdot X_{best}(t)-(G_{1}\cdot X(t)\cdot rand)-2(1-\frac{t}{T_{\max}})\cdot L+rand\cdot G_{1}$$

where *QF* is a quality function, and *G*_1_ denotes the hunting motion guidance. After each update, a Greedy Selection mechanism is applied, in which the position is updated only if the cost function is lower than the previous fitness. Finally, all candidate solutions are clipped to ensure the parameters remain within the feasible VLM discretization variable bounds.

## 3. Results and Discussions

### 3.1. Hybrid-RSM and ANOVA

**Figure 5:**
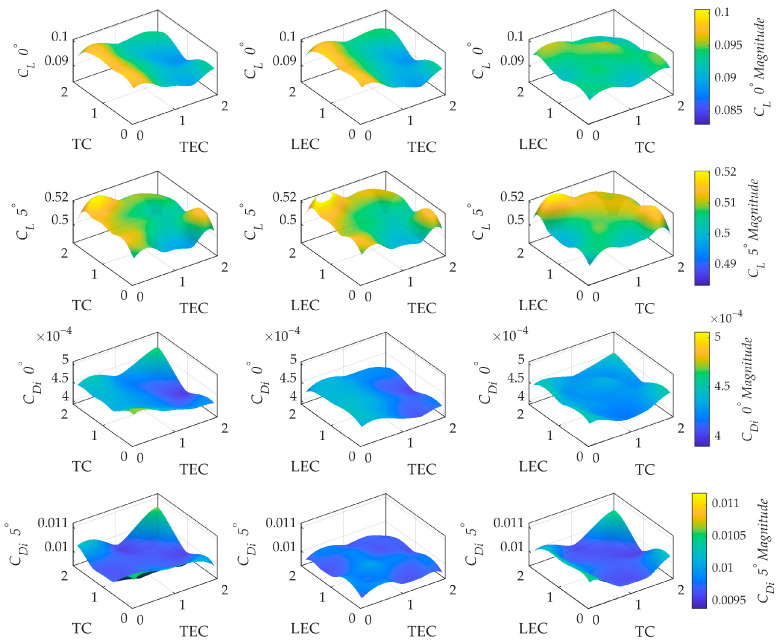
Hybrid response surface plots for the lift and induced drag coefficients with respect to TEC, LEC, and TC at 0° and 5° angles of attack.

The qualitative effects of discretization parameters on aerodynamic coefficients were examined using MATLAB (version R2023b), and the results are presented as three-dimensional RSM plots combining results from Box–Behnken and Central Composite designs in Figure 5. The surfaces were generated by evaluating the interaction-based model, enabling a direct comparison of spatial clustering effects on solution stability and providing a detailed overview of the VLM solver’s numerical sensitivity and response patterns.

For the lift coefficient at 0° angle of attack, the response surfaces showed a nearly flat topology with a steep slope only along the TEC axis. Such linear sensitivity indicates that the numerical solution is stable with respect to changes in leading edge or tip resolution, provided the Kutta condition is satisfied at the trailing edge. However, at 5° angle of attack, a notable topological transformation was observed. The surfaces for *C_L_* exhibited a unique multi-directional curvature, with the slope responding to variations in both LEC and TC. The appearance of curvature indicates a shift from a regime dominated by a single parameter to a multi-variable sensitivity state, reflecting the increased complexity of capturing the suction peak and spanwise distribution under higher lift conditions.

The surfaces for the induced drag coefficient exhibit different numerical characteristics. For instance, the response surface remains relatively flat at low magnitudes at 0° angle of attack and shows a slight bias toward trailing edge resolution. On the other hand, the surface exhibits a clear parabolic trend toward the TC axis at 5° angle of attack. The high-sensitivity zone is where high TEC and TC values occur, indicating that drag prediction requires a coordinated refinement approach. The surfaces have a smooth, consistent topology, free of irregular twists or unstable saddle points. Such a result suggests that the discretization parameters are compatible, collaborating to create a stable overall numerical trend rather than generating localized, unpredictable noise. The absence of these complex distortions indicates that the parameters interact stably.

Analysis of Variance was carried out to investigate the quantitative effects of VLM clustering parameters using the MATLAB Statistics and ML Toolbox at a 95% confidence level, and the results are presented in Table 2 and Table 3. The total variance was partitioned into the sum of squares for each individual factor and the residual error, allowing for the calculation of *F*-statistics and corresponding *p*-values.

For the lift coefficient at 0° angle of attack, TEC is identified as the only statistically significant parameter (*p* = 3.81 × 10^−14^) in Table 2, accounting for the vast majority of the model’s variance and confirming that the Kutta condition resolution is the main factor for analysis stability. As the angle of attack rises to 5°, the importance of LEC and TC emerges, as demonstrated by the greater curvature of the RSM surfaces. This emphasizes the necessity of accurately capturing the leading-edge suction peak and tip-vortex gradients.

The induced drag sensitivity demonstrated a certain shifting in terms of dominance in Table 3. TEC remains the most significant variable (*p* = 7.65 × 10^−14^) at 0° angle of attack, while TC became the dominant factor, with a highly significant *p*-value of 5.19 × 10^−12^ at 5° angle of attack. The case agrees with aerodynamic theory, as induced drag is directly proportional to the spanwise distribution of circulation, requiring dense resolution at the wingtips to capture the strong trailing vortices. The behavior of interaction terms yielding *p*-values higher than 0.05 across all cases clearly indicates that the discretization parameters act independently on the global force coefficients.

**Figure 6:**
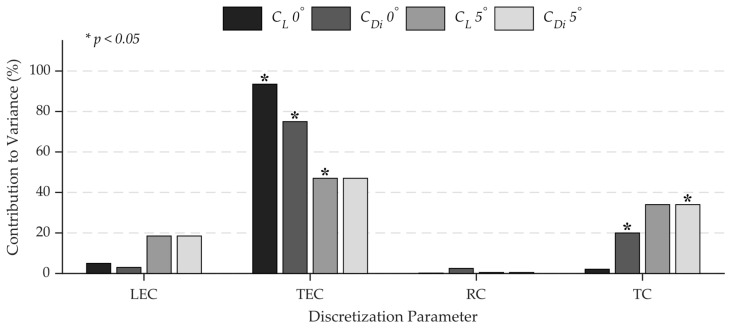
Contribution of discretization parameters to the variance of the lift and induced drag coefficients.

The statistical significance reported in the ANOVA tables is also supported visually by the variance decomposition shown in Figure 6, which quantifies the contribution of each discretization parameter to the overall numerical variance at different angles of attack.

The sharp reduction in dominance of TEC from 0° to 5° angle of attack indicates that the numerical error is mainly concentrated at the trailing edge at low angles of attack, while at higher lift conditions, the error shifts across the leading edge and wingtips. Such a result indicates that a panel discretization refinement approach designed for cruise conditions (i.e., at 0° angle of attack) would be ineffective for high-lift maneuvers, since the contributions of LEC and TC increase by about three times. The figure also emphasizes parameter-specific sensitivities; for example, *C_L_* stays relatively sensitive across all clustered regions at 5° angle of attack, while *C_Di_* is primarily influenced by the TEC:TC combination, with LEC having a secondary impact, proving that induced drag prediction is more sensitive to spanwise flow gradients than chordwise suction peaks. The minimal impact of RC highlights that root clustering refinement does not significantly improve accuracy; however, it efficiently reduces the optimization search space to the wing’s outer edges and chordwise extremities. Therefore, RC was excluded from the previously presented ANOVA and response surface analysis tables due to its mentioned negligible impact on the objective function.

### 3.2. Surrogate Model Generation

**Figure 7:**
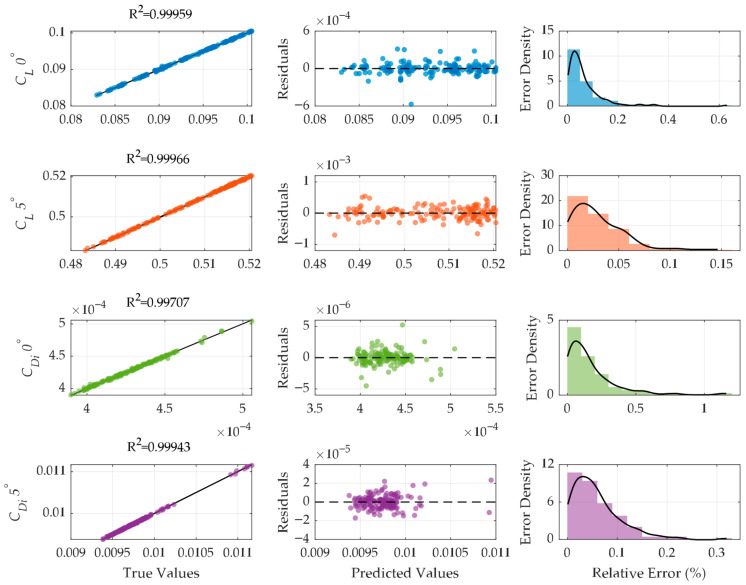
Regression, residual distribution, and error density results of the lift and induced drag coefficients at 0° and 5° angles of attack.

Surrogate models were generated to estimate the aerodynamic responses of the lift and induced drag coefficients at 0° and 5° angles of attack, providing the predictive framework for all objective function evaluations during the following optimization process. To generate models, an EML strategy based on LSBoost regression was adopted, as boosting methods are inherently more robust to localized nonlinearities and heterogeneous error distributions. The predictive performance of the trained surrogates is summarized in Figure 7 through parity, residual distribution, and error density results. To prevent overfitting, the learning rate and leaf size were carefully tuned, ensuring the high R^2^ values reflect true physical trends rather than data memorization. The dataset was not split into training and test sets and was used in its entirety to capture the full physics of the design space defined by the BBD and CCD matrices.

All four responses exhibit excellent agreement between predicted and reference values, with coefficients of determination above 0.997, demonstrating that the ensemble framework successfully captures the nonlinear relationship between discretization parameters and aerodynamic outputs. The parity plots show almost perfect alignment along the unity line, indicating both high accuracy and negligible systematic bias across the sampled design space. The residuals in the analysis confirm these findings, showing a symmetric distribution around zero for all outputs and no noticeable trends related to the predicted values. Such behavior indicates that the models lack structural bias and that heteroscedasticity remains minimal within the examined parameter ranges. Furthermore, the predictions of induced drag exhibit slightly greater residual dispersion than those of lift, suggesting that vortex-dominated drag mechanisms are more sensitive to discretization variations.

The probability density functions of relative errors exhibit pronounced right skew, with most predictions concentrated within a narrow range of errors. Lift coefficients exhibit very tight error clustering, whereas induced drag responses show broader tails, consistent with their greater numerical sensitivity to panel resolution and clustering strategies. Nevertheless, for drag-related outputs, the main error remains limited to small relative differences, confirming the surrogate’s robustness.

The regression accuracy of the developed LSBoost ensemble models demonstrated near-perfect correlation; however, to rigorously assess the models’ generalization capability and to ensure that the high predictive accuracy was not due to overfitting, a 10-fold cross-validation (CV) procedure was implemented. Table 4 presents a comparative analysis of the training and cross-validation performance metrics, including *R*^2^, Root Mean Square Error (RMSE) and Standard Deviation (Std).

The results indicate that the 10-fold CV *R*^2^ values remain remarkably high, with training and validation performance within a narrow margin, confirming the robustness of the ensemble learning approach. The RMSE values offer a more detailed view of the model’s accuracy. The CV-RMSE values for the lift coefficients indicate that the models can accurately capture subtle aerodynamic variations. The induced drag coefficient models show even greater sensitivity, with error margins as low as 10^−6^. The convergence of these metrics indicates that the LSBoost algorithm successfully models the underlying non-linear physics of the design space, rather than just interpolating the training data. The low CV RMSE Std values across all models indicate that the LSBoost algorithm is highly stable and consistent. This small variation across different data folds shows that the model’s predictive ability is not affected by specific data subsets, confirming that the high R^2^ values are statistically reliable and not due to random data splitting.

Consequently, the resulting ensemble models demonstrated significantly improved stability and predictive fidelity across all outputs, validating that LSBoost ensembles are reliable surrogates for replacing VLM evaluations in our optimization framework. providing sufficient sensitivity even for small variations in the aerodynamic coefficients.

#### Statistical Diagnostics and Predictive Performance Results

The robust performance and predictive reliability of the generated ensemble surrogate models rely on the statistical quality and proper distribution of the training data. In this study, a hybrid framework combining BBD and CCD was proposed to enhance the model’s generalization and enable more extensive exploration of the design space. While this combination increases sampling density, it requires careful validation to avoid biases arising from overlapping factor ranges and non-coincident design centers.

To provide a statistical basis for the applied hybrid approach, the design matrix was evaluated using key efficiency and orthogonality metrics. The condition number, D-efficiency, and average Variance Inflation Factors (VIF) were calculated for hybrid and individual models to assess and compare the numerical stability and multicollinearity of the combined design space, as detailed in Table 5.

The diagnostic results indicate that the hybrid design preserves strong structural integrity, noting that the final sample sizes (n) were obtained by subsequently eliminating intrinsic replicates within each design to ensure that the surrogate model was trained on a unique dataset, avoiding potential bias toward over-represented regions of the factor space. The average VIF of 1.0108 indicates that the correlation among input factors remains negligible, even with the higher point density in overlapping areas. This confirms that the higher data density in overlapping regions does not lead to multicollinearity; therefore, the surrogate model can estimate coefficients with high precision, overcoming the limitations typically encountered in unbalanced hybrid designs. Furthermore, the hybrid design exhibited condition numbers and D-efficiencies similar to those of the individual BBD and CCD frameworks, confirming its mathematical validity for high-dimensional surrogate modeling. The hybrid design also maintains a high degree of orthogonality, which ensures that the effects of individual factors can be estimated independently with minimal confounding. Consequently, the diagnostic metrics confirm that the hybrid approach effectively merges the geometric advantages of both BBD and CCD without compromising the design’s statistical efficiency.

**Figure 8:**
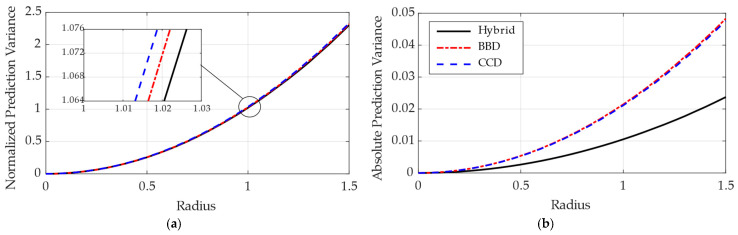
Variance dispersion graphs comparing the hybrid RSM design with BBD and CCDs: (**a**) normalized prediction variance; (**b**) absolute prediction variance as a function of design radius.

The predictive reliability of the proposed hybrid scheme was further examined using Variance Dispersion Graphs (VDG), as shown in Figure 8.

While the normalized prediction variance in Figure 8a remains relatively consistent across all methodologies, the analysis of absolute prediction variance in Figure 8b highlights a key benefit of the hybrid approach. The hybrid design reduced the absolute variance by approximately 50% compared to the individual BBD and CCDs, which demonstrates substantially lower prediction uncertainty throughout the design radius, particularly in the outer regions where individual designs typically exhibit greater variance. This improvement is attributed to the strategic placement of sample points in both the spherical sub-regions of the BBD and the axial or corner regions of the CCD. Such a significant reduction provides a more reliable data foundation, enabling the ensemble surrogate model to achieve superior accuracy and robustness throughout the entire explored factor space.

### 3.3. Optimization Results

The trained ensemble surrogate models were integrated into the optimization framework to identify the best panel clustering parameters (*LEC*, *TEC*, *RC*, *TC*) that minimize the error between VLM simulations at 0° and 5° angles of attack and experimental data. In a comparative perspective, the bio-inspired AO and COA were evaluated alongside the GA as a baseline, utilizing a population of 30 over 500 iterations. Each stochastic algorithm was tested through 20 independent runs to ensure statistical reliability.

**Figure 9:**
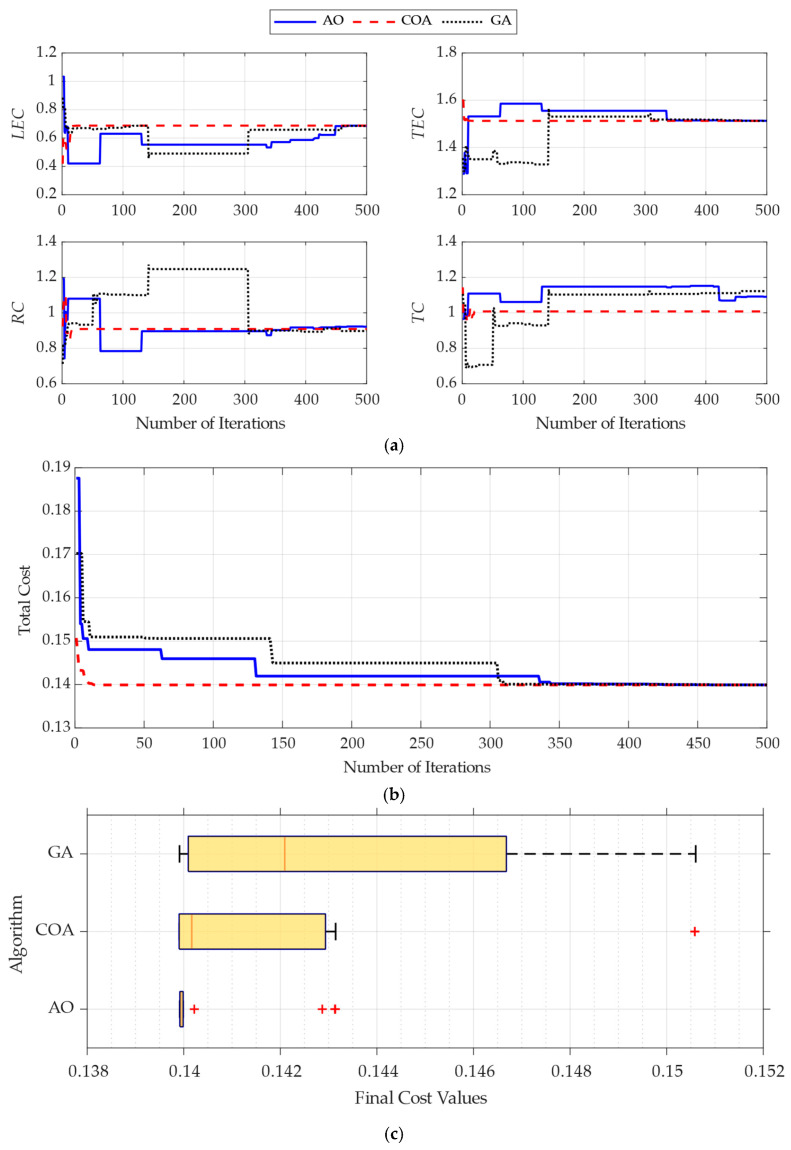
Optimization performance of AO, COA, and GA: (**a**) evolution of design variables, (**b**) convergence of the total cost function, and (**c**) statistical distribution of final solutions.

The optimization performance of AO and COA in terms of the evolution of design variables, convergence patterns, and solution robustness over iterations is demonstrated in Figure 9. As shown in Figure 9a, both algorithms quickly adjusted the design variables from the early iterations, indicating effective global exploration. COA achieved stable parameter values earlier, indicating quicker exploitation, whereas AO kept refining variables over a longer search duration through iterations, illustrating a better balance between exploration and exploitation. The convergence histories in Figure 9b show that both algorithms achieved nearly the same minimum cost values. However, COA converged quickly initially, whereas AO exhibited a more gradual and consistent decrease in the objective function. This indicates that AO maintained search diversity for a longer period, enabling further improvements in later iterations. Additionally, the statistical distribution of final solutions across multiple independent runs is shown in Figure 9c. AO results are in a much narrower distribution, indicating greater robustness and repeatability. In contrast, COA showed greater variability, even though it achieved similar best-cost values.

Table 6 summarizes the comparative statistical results, showing that both algorithms find the global minimum cost of 0.13991. However, their stability and convergence reliability differ notably because of their distinct bio-inspired frameworks. The AO demonstrated exceptional accuracy, with its mean and median values aligning perfectly with the optimal cost, as shown by a very low standard deviation. This stability is attributed to the short-glide and narrow-descent attack phase of the eagle, which helps the algorithm focus its search with deterministic accuracy once the optimal region for panel clustering is located. Conversely, the COA showed a much higher standard deviation and a worst-case cost, indicating a more scattered performance profile. This behavior arises from crayfish’s heat-avoidance and food competition mechanisms; while these random movements are useful for exploring a broad area, they also create unpredictability, making consistent fine-tuning during the final exploitation stages more challenging. Consequently, AO’s targeted hunting strategy proved more reliable than COA’s randomized foraging approach, ensuring that the same high-fidelity discretization parameters are achieved across independent trials.

Success rate (SR) is defined as the percentage of runs reaching a final cost within a 0.5% proximity to the best-known minimum (*f* ≤ 0.140605). The assessment of success rates across 20 independent trials evaluating operational robustness achieved a remarkable 85% success rate for AO, consistently identifying the global optimum in most of the runs. In contrast, the COA achieved only a 55% success rate, often getting trapped in local minima, as shown by its higher standard deviation in Table 6 and graphical distribution of the solutions in Figure 9c. This performance difference confirms that, for the complex search space of panel discretization, the AO algorithm offers an extremely reliable framework, ensuring high-quality results regardless of stochastic initialization.

The source of the complexity in this optimization problem that limits both algorithms’ ability to achieve 100% success is not the dimensionality (with four variables) but rather the inherent topological nature of the generated surrogate model. Although the LSBoost ensemble model achieves very high predictive accuracy (R^2^ > 0.997), it does not produce a smooth or continuous response surface because LSBoost partitions the search space into orthogonal regions, a fundamental characteristic of regression trees, which results in a piecewise-constant, non-differentiable landscape featuring tiny flat regions and abrupt, step-like discontinuities. As stochastic agents navigate this rugged terrain and land on these artificial flat regions, they encounter zero local gradients. This complete lack of gradient information heavily causes premature convergence, leading the algorithms to get stuck in the surrogate model’s shallow local minima. In this highly misleading context, the differences in performance can be clearly attributed to the underlying biomimetic mechanisms of the algorithms. The 85% success rate of AO highlights its strong exploration abilities, which mathematically simulate the high-altitude soaring and wide visual field of an Aquila. This global, ‘bird’s-eye’ search strategy allows the AO agents to effectively see beyond the localized flat regions and jump over the piecewise plateaus. In contrast, the COA replicates the crayfish’s localized, bottom-foraging, and crawling behaviors. Despite the algorithm’s effectiveness in smooth environments, using ‘crawling’ to traverse a stepped, tree-based surrogate landscape makes COA agents highly prone to zero-gradient traps, reducing their success rate. Similarly, the traditional GA, without such dynamic spatial exploration strategies, produced the lowest success rate, further demonstrating the need for advanced global search methods when working with tree-based surrogate topographies.

To assess the practical applicability of the proposed methodology at early conceptual design stages, a comprehensive time-cost analysis was conducted, encompassing both offline data generation (HRSM data sampling) and optimization iterations. The computational time (CT) breakdown is presented in Table 7.

As observed, generating the aerodynamic dataset using the VLM solver for 97 unique panel discretization configurations required a one-time computational effort of 228.7 min (~2.35 min per VLM evaluation), while training the LSBoost surrogate took less than a minute. For robust statistical validation across 20 independent runs, the cumulative CPU effort was approximately 42 min per algorithm. However, the framework can achieve optimal panel discretization in approximately 4 h, with optimization enabled via parallel processing. This computational effort represents a highly beneficial trade-off, as it ensures high aerodynamic accuracy early in the conceptual design phase, since the literature widely establishes that 70% to 80% of an aerospace vehicle’s total life-cycle cost is committed during the conceptual design stage [41]. By ensuring high aerodynamic accuracy early in the design, the framework avoids major misjudgments in configuration and protects the development timeline from costly late-stage revisions, requiring only a small initial time investment.

Table 8 provides the best design variables and a detailed overview of the statistical strength of the algorithms and their alignment with the resulting optimal design parameters. With respect to the initial uniform discretization, the optimized panel clustering configuration achieved a 32.98% reduction in total cost.

A comparative analysis was conducted between single-point optimization strategies (targeting *C_L_* and *C_Di_* at only 0° or 5° angle of attack) and the proposed combined-objective approach to assess the effectiveness of a multi-objective method. The performance trade-off analysis results achieved using AO for various objective targets are summarized in Table 9. The findings show that although a panel discretization optimized for the 0° angle of attack (AoA) condition yields high local accuracy, its performance drops noticeably at 5° AoA. This indicates that a grid optimized for low-angle cruise conditions lacks sufficient resolution to accurately capture the more complex pressure gradients and lift-induced flow physics that occur at higher AoA. Conversely, the discretization optimized for 5° angle of attack shows a poor generalization to the 0° condition. These differences reveal that optimizing at a single point often overfits the discretization topology to a particular flow regime, undermining overall reliability.

**Figure 10:**
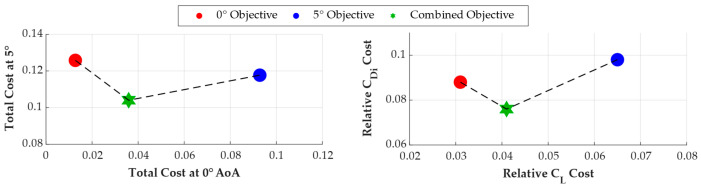
Evaluation of the optimized panel discretization within the Pareto-optimal fronts for different AoA objectives.

The combined strategy successfully balances these trade-offs by identifying an optimal discretization setup, as illustrated in Figure 10. The combined objective serves as a numerical regularizer, preventing the optimizer from converging to extreme parameter values that would favor a single state. The strategy finds a solution at the “knee” of the Pareto front, where discretization errors for both 0° and 5° conditions are minimized simultaneously without significantly compromising one for the other. Furthermore, the method results in a 5° cost that is even lower than the outcome of the 5° objective optimization. This indicates that including the 0° data in the objective function helps the optimizer avoid local minima and guides for a more globally optimal grid topology. By balancing LEC and TEC parameters, the combined strategy ensures high-fidelity resolution of both the leading-edge suction peaks and the trailing-edge wake recovery, which are critical for accurate predictions of lift and drag. In conclusion, the weighted multi-point objective strategy has been confirmed to produce a Pareto-optimal panel discretization.

**Figure 11:**
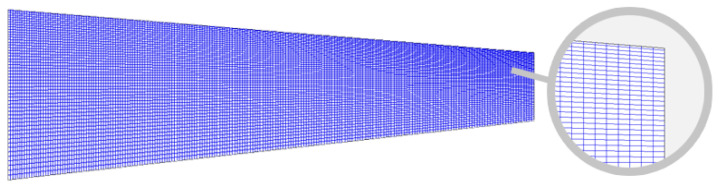
Top view of the half-wing geometry with optimized panel discretization.

Figure 11 illustrates the wing geometry discretized with optimized panel clustering parameters. The resulting grid has dense panels in aerodynamically significant regions. Specifically, the reduction in LEC and TC indicates that a higher panel density is required at the leading edge and wing tip to accurately resolve high-gradient flow regions. The optimization maintained a higher TEC, suggesting that a relatively coarser distribution near the trailing edge is sufficient when the Kutta condition is properly satisfied. The stability of the RC near the unit value, combined with its negligible sensitivity in ANOVA, confirms that the flow field at the wing root is less sensitive to panel density in the absence of fuselage interference. These results demonstrate that the optimization process went beyond just reducing a mathematical error; it also effectively pinpointed the key aerodynamic regions where high panel resolution is essential for accurately representing physical phenomena. This adaptive method shows how bio-inspired optimization successfully reallocated panels in response to local flow sensitivity.

The quantitative comparison between VLM predictions for optimized and base uniform discretizations is summarized in Table 10, along with experimental data. The optimized configuration significantly improved aerodynamic accuracy, especially at low angles of attack, reducing relative errors in *C_L_* at 0° and *C_Di_* at 5°. At 5° angle of attack, *C_Di_* exhibited a modest improvement, while the lift coefficient showed a slight increase in relative error. Similarly, the lift curve slope showed a slight variation relative to uniform discretization, remaining within a similar error range. Such behavior illustrates a necessary trade-off in multi-point optimization: the algorithms focus on minimizing induced drag error, which is crucial for performance estimation in potential flow solvers, rather than a slight deviation in lift at higher angles of attack. Therefore, while the overall error is reduced, the sensitivity of the lift coefficient at higher angles indicates the redistribution of discretization error rather than a decrease in model accuracy.

These results indicate that the optimized panel clustering primarily enhanced VLM accuracy in low-angle regimes and induced drag prediction, while higher-angle lift characteristics remained sensitive to discretization and model limitations.

**Figure 12:**
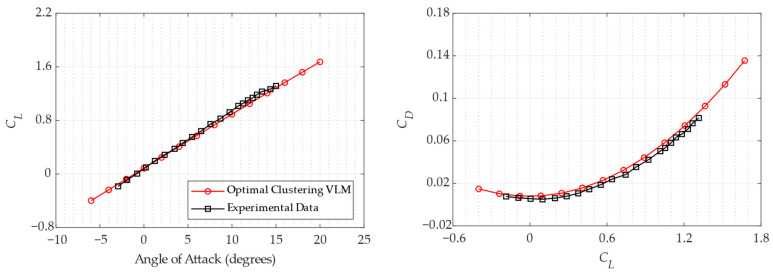
Aerodynamic validation of optimized panel clustering: comparison of VLM predictions with experimental data.

Finally, the VLM analysis using optimal clustering options was carried out within a wide range of angles of attack, and the results are presented in Figure 12 as lift-curve and drag-polar comparisons with experimental data.

The lift curve, with a mean squared error (MSE) of 1.8 × 10^−3^, indicates that the optimized panel distribution accurately captures the slope, effectively bridging the gap between the initial uniform discretization and the experimental data. Additionally, the drag polar with MSE of 5.13 × 10^−6^ shows that the induced drag components, which are highly sensitive to panel density near the leading and trailing edges, are more accurately resolved. While the VLM naturally lacks viscous drag modeling, leading to a slight offset at higher lift coefficients, the optimized clustering reduced numerical errors in the inviscid component and provided a superior performance for initial design and optimization.

The error margins for both lift and drag components are consistent with the inherent limitations of potential flow theory documented in the literature, suggesting that remaining deviations are mainly due to physics modeling rather than discretization issues. The decrease in the error in especially low angles of attack for *C_L_*_0_ to 3.17% and *C_Di_*_0_ to 3.54% indicates a highly accurate result for a quasi-linear solver. In similar studies in the literature using traditional VLM or low-order panel methods with/without discretization, error margins for both lift and drag coefficients are typically higher than those achieved in this study with an optimization strategy [30,42,43,44]. In this context, the main strength of this study is its ability to overcome uniform discretization by using a novel bio-inspired metaheuristic-driven framework, whereas common practices rely on manual cosine spacing or empirical clustering. Such a degree of precision is similar to that achieved by higher-order methods or carefully calibrated Computational Fluid Dynamics simulations in linear regimes, yet it requires significantly less computational effort.

## 4. Conclusions

This study presents a case study on a comprehensive bio-inspired optimization framework for VLM panel discretization, integrating HRSM, ensemble machine-learning surrogates, and Crayfish–Aquila optimization algorithms. A hybrid Box–Behnken–Central Composite experimental design was employed to maximize design space exploration with minimal sampling, systematically exploring clustering parameters in the leading-edge, trailing-edge, root, and tip regions of a representative tapered wing geometry, while ensuring solution independence through grid-convergence analysis.

For the investigated wing configuration, the optimized discretization enhanced overall aerodynamic prediction accuracy by 32.98%, with especially significant improvements at low angles of attack and consistent performance at higher angles of attack. The optimized discretization showed exceptional agreement with experimental data in the linear aerodynamic regime (low angles of attack); its predictive accuracy naturally declined at higher angles due to the absence of viscous modeling. This reinforces the proposed framework’s position as a high-fidelity tool for conceptual and preliminary design stages specifically where inviscid flow assumptions remain valid.

Bio-inspired optimizers both converged to similar minima within the surrogate-based landscape; however, Aquila achieved 85% global convergence, while Crayfish converged faster but with greater dispersion, suggesting a multimodal optimization landscape inherent to the surrogate-modeled discretization space. The stability of AO is attributed to its deterministic, targeted hunting phase, which effectively fine-tunes sensitive discretization variables.

ANOVA-based variance decomposition revealed that trailing-edge clustering dominates aerodynamic accuracy at low angles of attack, contributing up to 90% of the total variance for this tapered wing topology. This result aligns with the enforcement of the Kutta condition in the VSPAERO horseshoe-vortex formulation. Conversely, tip clustering becomes increasingly important at higher angles, exceeding 30%. The optimized values align with aerodynamic theory, underscoring the need for dense paneling in regions dominated by suction peaks and tip vortices to accurately capture physical reality of the investigated wing. The RSM combined with ANOVA statistics provided a robust basis for panel discretization optimization.

The outcomes of this case study demonstrate that adaptive, performance-driven panel clustering offers a physically meaningful alternative to heuristic discretization strategies, enabling enhanced VLM accuracy without increasing computational complexity. Furthermore, the superior consistency of the AO suggests that, in high-dimensional aerodynamic search spaces, deterministic targeted exploitation is more valuable than purely stochastic exploration. This finding can guide future studies in choosing optimizers for more complex multidisciplinary design optimization tasks. Consequently, the proposed innovative hybrid RSM–ML–optimization framework provided an efficient and scalable approach for the early stages of aircraft design, offering a systematic procedure that can be extended to evaluate more complex geometries and multidisciplinary applications. Future research will aim to expand this framework to more complex aerodynamic topologies, such as high-sweep delta wings and multi-element lifting surfaces, where the sensitivity of discretization parameters is likely to vary considerably. In addition, incorporating viscous effects and structural constraints into the optimization process could further improve the framework’s robustness for comprehensive multidisciplinary design optimization (MDO) tasks.

## Figures and Tables

**Table 1:** Design variables and their levels used in the design of experiments.

Variable	Minimum		Center		Maximum	
	BBD	CCD	BBD	CCD	BBD	CCD
TEC	0.1	0.575	0.8	1.05	1.5	1.525
LEC	0.1	0.575	0.8	1.05	1.5	1.525
TC	0.1	0.575	0.8	1.05	1.5	1.525
RC	0.1	0.575	0.8	1.05	1.5	1.525

**Table 2:** ANOVA summary and statistical significance of clustering parameters for lift coefficient.

Factor	Sum of Squares (SS)		Mean Square (MS)		*F*-Value		*p*-Value	
	*C_L_* 0°	*C_L_* 5°	*C_L_* 0°	*C_L_* 5°	*C_L_* 0°	*C_L_* 5°	*C_L_* 0°	*C_L_* 5°
TEC	0.0010	0.0013	0.0010	0.0013	70.71	12.99	3.81 × 10^−14^	4.31 × 10^−4^
TC	7.02 × 10^−6^	6.86 × 10^−4^	7.01 × 10^−6^	6.86 × 10^−4^	0.472	6.66	0.492	0.010
LEC	5.49 × 10^−5^	6.05 × 10^−4^	5.49 × 10^−5^	6.05 × 10^−4^	3.702	5.874	0.056	0.016
TEC:TC	1.49 × 10^−6^	3.63 × 10^−5^	1.49 × 10^−6^	3.63 × 10^−5^	0.100	0.352	0.751	0.553
TEC:LEC	4.31 × 10^−8^	5.90 × 10^−6^	4.31 × 10^−8^	5.90 × 10^−6^	0.002	0.057	0.957	0.811
TC:LEC	3.21 × 10^−7^	1.59 × 10^−7^	3.21 × 10^−7^	1.59 × 10^−7^	0.021	0.001	0.883	0.968
Residuals	0.0019	0.0146	1.48 × 10^−5^	1.03 × 10^−4^	1	1	0.500	0.500

**Table 3:** ANOVA summary and statistical significance of clustering parameters for induced drag coefficient.

Factor	Sum of Squares (SS)		Mean Square (MS)		*F*-Value		*p*-Value	
	*C_Di_* 0°	*C_Di_* 5°	*C_Di_* 0°	*C_Di_* 5°	*C_Di_* 0°	*C_Di_* 5°	*C_Di_* 0°	*C_Di_* 5°
TEC	1.85 × 10^−8^	2.11 × 10^−7^	1.85 × 10^−8^	2.11 × 10^−7^	68.66	2.75	7.65 × 10^−14^	0.098
TC	2.44 × 10^−9^	4.34 × 10^−6^	2.44 × 10^−9^	4.34 × 10^−6^	9.04	56.79	0.0031	5.19 × 10^−12^
LEC	4.91 × 10^−10^	1.12 × 10^−7^	4.91 × 10^−10^	1.12 × 10^−7^	1.820	1.468	0.179	0.227
TEC:TC	3.42 × 10^−10^	7.75 × 10^−8^	3.42 × 10^−10^	7.75 × 10^−8^	1.267	1.012	0.262	0.316
TEC:LEC	3.81 × 10^−11^	1.06 × 10^−8^	3.81 × 10^−11^	1.06 × 10^−8^	0.141	0.139	0.707	0.709
TC:LEC	1.34 × 10^−11^	1.15 × 10^−8^	1.34 × 10^−11^	1.15 × 10^−8^	0.049	0.150	0.824	0.698
Residuals	3.86 × 10^−8^	1.08 × 10^−5^	2.70 × 10^−10^	7.65 × 10^−8^	1	1	0.500	0.500

**Table 4:** Comparison of training and 10-fold cross-validation performance.

Model	Training R^2^	10-Fold CV R^2^	Training RMSE	10-Fold CV RMSE	Std
*C_L_*_0_	0.9995	0.9789	9.39 × 10^−5^	6.61 × 10^−4^	2.54 × 10^−4^
*C_L_*_5_	0.9996	0.9509	1.09 × 10^−6^	4.78 × 10^−6^	5.55 × 10^−4^
*C_Di_*_0_	0.9970	0.9828	1.98 × 10^−4^	1.28 × 10^−3^	2.12 × 10^−6^
*C_Di_*_5_	0.9994	0.9765	7.82 × 10^−6^	5.01 × 10^−5^	1.64 × 10^−5^

**Table 5:** Comparative statistical design diagnostics and efficiency metrics for Hybrid, BBD, and CCDs.

Method	*n*	Condition Number	D-efficiency	Orthogonality (%)	VIF (Avg.)
Hybrid	97	1.1441	0.2789	93.89	1.0108
BBD	48	1.1224	0.2782	94.95	1.0083
CCD	49	1.1767	0.2793	92.89	1.0149

**Table 6:** Comparative statistical performance of AO, COA, and GA over multiple runs.

Algorithm	Best (Min)	Worst (Max)	Mean	Median	Std. Deviation
GA	0.139912	0.150602	0.143582	0.142091	0.00410266
AO	0.139916	0.143139	0.140420	0.139939	0.00113462
COA	0.139906	0.150584	0.141990	0.140165	0.00323207

**Table 7:** Computational time required for the process across the algorithms.

Algorithm	Mean CT for a Run (min)	Total CT (min)	Surrogate Model Training (min)	HRSM Data Sampling (min)
GA	2.08	41.66 *	<1	228.7
AO	2.13	42.56 *		
COA	2.12	42.37 *		

* The average wall-clock time for the parallelized execution of the 20 independent optimization trials is approximately 18.5 min.

**Table 8:** Optimal design parameters and minimum cost values achieved by AO, COA and GA.

Algorithm	LEC	TEC	RC	TC	Final Cost	Base Cost
GA	0.6853	1.5127	0.8977	1.1227	0.13991	0.22284
AO	0.6855	1.5128	0.9220	1.0907	0.13992	
COA	0.6875	1.5125	0.9090	1.0082	0.13991	

**Table 9:** Performance trade-off analysis for different angles of attack objectives.

Objective AoA	LEC	TEC	RC	TC	0° Cost	5° Cost
0°	0.6494	1.5126	0.8614	0.9261	0.012749	0.125765
5°	0.6850	0.8462	0.6871	1.2776	0.092834	0.117675
Combined	0.6855	1.5128	0.9220	1.0907	0.035900	0.104010

**Table 10:** Comparison of VLM results using uniform and optimized panel clustering against experimental data.

Variable	Base Uniform	Difference with Exp. (%)	Optimal Clustering	Difference with Exp. (%)	Experimental Data
*C_L_*_0_	0.08812	7.46	0.08460	3.17	0.082
*C_Di_*_0_	0.0004145	6.28	0.0004038	3.54	0.00039
*C_L_*_5_	0.49491	3.33	0.4890	4.49	0.512
*C_Di_*_5_	0.009630	12.45	0.009696	11.85	0.011
*∂C_L_*/*∂α* (*deg*)	0.08135	5.39	0.08088	5.94	0.08599

## Data Availability

The original contributions presented in this study are included in the article. Further inquiries can be directed to the corresponding author.

## Supplementary Materials

The following supporting information can be downloaded at: https://www.mdpi.com/article/10.3390/biomimetics11030204/s1, Table S1: Box–Behnken experimental design; Table S2: Central composite experimental design.
