# Supplementary material for: Parametric Optimization of VLM Panel Discretization Using Bio-Inspired Crayfish and Aquila Algorithms Coupled with Hybrid RSM-Based Ensemble Machine Learning Surrogate Models: A Case Study
Source: Biomimetics (Basel). 2026 Mar 11;11(3):204. doi: 10.3390/biomimetics11030204 (PMC13023603; doi:10.3390/biomimetics11030204)
Supplement: Supplementary file 1 [file biomimetics-11-00204-s001.zip › biomimetics-4154043-supplementary.pdf]

biomimetics

MDPI

Table S1. Box-Behnken experimental design.

RunOrder	StdOrder	PtType	LEC	TEC	RC	TC
1	9	2	0.1	0.8	0.8	0.1
2	11	2	0.1	0.8	0.8	1.5
3	19	2	0.1	0.8	1.5	0.8
4	18	2	1.5	0.8	0.1	0.8
5	12	2	1.5	0.8	0.8	1.5
6	27	0	0.8	0.8	0.8	0.8
7	23	2	0.8	0.1	0.8	1.5
8	15	2	0.8	0.1	1.5	0.8
9	3	2	0.1	1.5	0.8	0.8
10	6	2	0.8	0.8	1.5	0.1
11	17	2	0.1	0.8	0.1	0.8
12	24	2	0.8	1.5	0.8	1.5
13	1	2	0.1	0.1	0.8	0.8
14	8	2	0.8	0.8	1.5	1.5
15	21	2	0.8	0.1	0.8	0.1
16	16	2	0.8	1.5	1.5	0.8
17	25	0	0.8	0.8	0.8	0.8
18	26	0	0.8	0.8	0.8	0.8
19	7	2	0.8	0.8	0.1	1.5
20	10	2	1.5	0.8	0.8	0.1
21	4	2	1.5	1.5	0.8	0.8
22	2	2	1.5	0.1	0.8	0.8
23	14	2	0.8	1.5	0.1	0.8
24	22	2	0.8	1.5	0.8	0.1
25	5	2	0.8	0.8	0.1	0.1
26	20	2	1.5	0.8	1.5	0.8
27	13	2	0.8	0.1	0.1	0.8

Table S2. Central composite experimental design.

RunOrder	StdOrder	PtType	LEC	TEC	RC	TC
1	15	1	1.525	0.575	1.525	1.525
2	28	0	1.050	1.050	1.050	1.050
3	27	0	1.050	1.050	1.050	1.050
4	23	-1	1.050	1.050	0.100	1.050
5	21	-1	1.050	1.050	1.050	0.100
6	8	1	1.525	1.525	0.575	1.525
7	6	1	0.575	1.525	0.575	1.525
8	19	-1	0.100	1.050	1.050	1.050
9	12	1	1.525	1.525	1.525	0.575
10	30	0	1.050	1.050	1.050	1.050
11	11	1	1.525	0.575	1.525	0.575
12	2	1	0.575	1.525	0.575	0.575
13	16	1	1.525	1.525	1.525	1.525
14	4	1	1.525	1.525	0.575	0.575
15	24	-1	1.050	1.050	2.000	1.050
16	1	1	0.575	0.575	0.575	0.575

 https://doi.org/10.3390/biomimetics11030204

17	7	1	1.525	0.575	0.575	1.525
18	25	0	1.050	1.050	1.050	1.050
19	3	1	1.525	0.575	0.575	0.575
20	5	1	0.575	0.575	0.575	1.525
21	29	0	1.050	1.050	1.050	1.050
22	22	-1	1.050	1.050	1.050	2.000
23	26	0	1.050	1.050	1.050	1.050
24	18	-1	1.050	2.000	1.050	1.050
25	10	1	0.575	1.525	1.525	0.575
26	20	-1	2.000	1.050	1.050	1.050
27	17	-1	1.050	0.100	1.050	1.050
28	9	1	0.575	0.575	1.525	0.575
29	13	1	0.575	0.575	1.525	1.525
30	31	0	1.050	1.050	1.050	1.050
31	14	1	0.575	1.525	1.525	1.525
